# Proinflammatory IL-17 pathways dominate the architecture of the immunome in pediatric refractory epilepsy

**DOI:** 10.1172/jci.insight.126337

**Published:** 2019-04-18

**Authors:** Pavanish Kumar, Derrick Chan Wei Shih, Amanda Lim, Bhairav Paleja, Simon Ling, Lai Li Yun, Su Li Poh, Adeline Ngoh, Thaschawee Arkachaisri, Joo Guan Yeo, Salvatore Albani

**Affiliations:** 1 Translational Immunology Institute, Singhealth/Duke-NUS Academic Medical Centre, Singapore.; 2 Paediatrics Academic Clinical Programme, KK Women’s and Children’s Hospital, Singapore, Singapore.; 3 Duke-NUS Medical School and Rheumatology and Immunology Service, KK Women’s and Children’s Hospital, Singapore, Singapore.

**Keywords:** Immunology, Neuroscience, Cellular immune response, Epilepsy, T cells

## Abstract

Drug refractory epilepsy (RE) is a chronic neurological disease with varied etiology that represents a group of patients whose seizures do not respond to antiepileptic drugs. The immune system may have a role in seizure and epilepsy development, but the specific mechanisms of inflammation that lead to epileptogenesis and contribute to RE are unknown. Here, we used mass cytometry to comprehensively study the immune system of pediatric patients with RE and compared their immune profile and function with patients with age-matched autoimmune encephalitis (AIE) and healthy controls. Patients with RE and AIE displayed similar immune profiles overall, with changes in CD4^+^ and CD8^+^ T cell subsets and an unbalance toward proinflammatory IL-17 production. In addition, patients with RE uniquely showed an altered balance in NK cell subsets. A systems-level intercellular network analysis identified rewiring of the immune system, leading to loss of inhibitory/regulatory intercellular connections and emergence of proinflammatory pathogenic functions in neuroinflammatory immune cell networks in patients with AIE and RE. These data underscore the contribution of systemic inflammation to the pathogenesis of seizures and epileptogenesis and have direct translational implications in advancing diagnostics and therapeutics design.

## Abstract 2

The architecture of the immunome in pediatric refractory epilepsy is dominated by a emergence of pro-inflammatory, IL-17 dependent pathways.

## Introduction

Epilepsy affects more than 50 million people worldwide, and onset typically occurs in childhood. Epileptic seizures are initiated as unprovoked seizures in affected patients, arising from uncontrolled electrical activity in the brain. Conventional anticonvulsants that largely target ion channels only improve seizure control and cannot directly prevent epileptogenesis. Refractory epilepsy, or drug refractory epilepsy (RE), is defined as a failure of 2 appropriate and adequate antiepileptic drugs to control seizures. RE occurs in 30% of epilepsy cases and is associated with notable cognitive, behavioral, and socioeconomic difficulties. Autoimmune encephalitis (AIE) is an increasingly recognized cause of encephalopathy in children. AIE is often triggered by infections, and patients develop seizures as part of their clinical presentation. About 40% of AIE disorders are associated with pathogenic neuronal autoantibodies (1). Patients who recover from AIE often remain affected (10%–15% of AIE patients) by epilepsy, which in many cases is refractory (2). Although AIE responds well to immunotherapy and children with epileptic encephalopathies and refractory seizures are treated with steroids, there is little evidence for the efficacy of corticosteroids in RE as a whole (3, 4). The mechanism of action for corticosteroids in AIE is unclear; the therapeutic effects are postulated to be due to direct neuronal effects, metabolic changes, and immune-modulation or suppression. Recent studies have shown association of inflammation with seizures and epileptogenesis, even in the absence of an underlying inflammatory disorder (5–8). Animal models of epilepsy show inflammation and a perturbed immune response, and inflammation has been identified in surgically resected brain tissue from human patients with RE (8–12). Consequently, antiinflammatory molecules are now being evaluated in animal models for their antiepileptic effects (13). Rasmussen’s encephalitis — a chronic inflammatory brain disorder — predominantly affects children with onset of frequent seizure that often progress to RE. T cell receptor (TCR) repertoire analysis have shown sharing of TCR clones between the infiltrating brain and peripheral T cells, clearly suggesting the role of T cells in epilepsy development (10, 12). In an elegant humanized mouse engraftment study, Kebir et al. has demonstrated that trafficking of patient T cells from periphery to mouse brain causes seizure in the mouse (7). These studies clearly demonstrate the involvement of peripherally derived T cells in seizure and epileptogenesis. Deepening our understanding of the immune response and inflammatory mediators involved in epilepsy and similar neurological disorders will enhance our understanding of epileptogenesis and lead to precise targets for therapeutic intervention.

Here, we aimed to unravel the intricate balance and contribution of various immune cells to the inflammatory profile of patients with RE and AIE. We analyzed immune function at a systems level using a high-dimensional single-cell technology **—** cytometry by TOF (CyTOF) **—** that permits >40 parameters to be analyzed simultaneously. We hypothesized that the distribution of immune subsets might be unique to different pathophysiological conditions and could differentiate healthy controls from patients with neuroinflammatory (NI) epileptic conditions. By coupling CyTOF with an unbiased data-driven analysis approach, we identified unique subsets of immune cells that are dysregulated in RE and AIE. Specifically, we observed an expansion of proinflammatory CD4^+^ and CD8^+^ T cells and a contraction of inhibitory receptors (LAG3 and PD1) positive CD8^+^ T cells. We also identified unique NK cell subsets specifically associated with RE. Importantly, our systems-level immune cell network analysis showed a dysregulation of the immune architecture toward loss of regulatory mechanisms and emergence of proinflammatory T cell subsets perpetuating chronic inflammation in RE.

## Results

### Major immune cell subsets are dysregulated in patients with RE and AIE.

**Figure 1:**
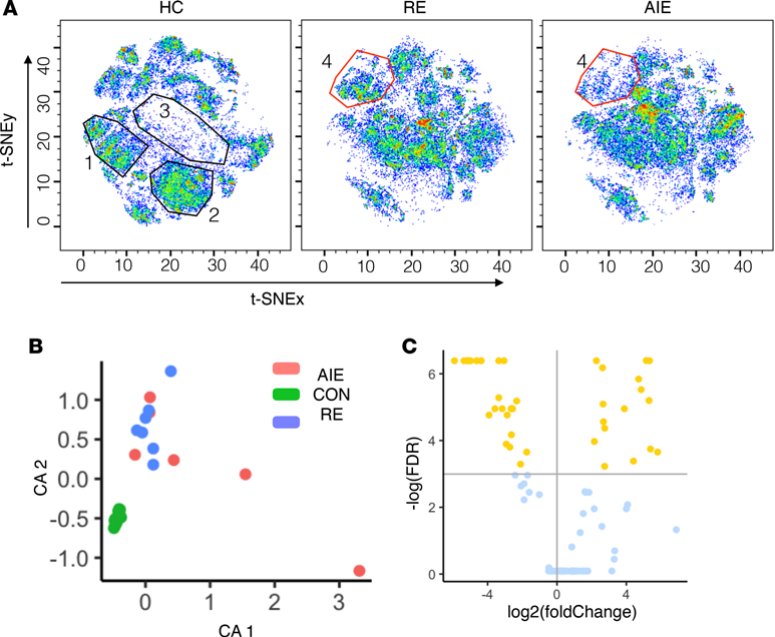
Data-driven analysis of the immunome shows major immune cell subset dysregulation in patients with neuroinflammatory epileptic disease. (**A**) Cell subset distribution was visualized on a t-SNE map. Separate t-SNE maps for healthy controls (HC), refractory epilepsy (RE), and autoimmune encephalitis (AIE) groups were plotted. The *x* and *y* axes correspond to the t-SNEx and t-SNEy coordinates, respectively. Each cell is plotted as a dot, and the color indicates the density of cells. Blue dots and empty spaces indicate low cell densities, while green and red dots indicate high cell densities. The t-SNE plot was visualized in pseudo-color using FlowJo software. (**B**) Correspondence analysis (CA) to summarize the distribution of immune cell subsets (clustered using k-means clustering algorithms) as 2 principal components, CA1 and CA2, for each individual from the HC, RE, and AIE groups. (**C**) Volcano plot shows the result of statistical comparison between HC and NI group. The *x* axis shows the fold change (log_2_ scale), and the *y* axis shows the FDR values (–log[FDR]). Nodes above the horizontal line (yellow dots) indicate significantly altered nodes with a 10% FDR cutoff. Nodes left of the vertical line are reduced in frequency, while nodes on the right are increased in frequency.

As shown in CyTOF staining, acquisition and analysis workflow (Supplemental Figure 1; supplemental material available online with this article; https://doi.org/10.1172/jci.insight.126337DS1), we first used CyTOF followed by 2-dimensional (2-D) t-distributed stochastic neighbor embedding (t-SNE) (14) to create a global profile of immune cells in our 3 groups Unlike conventional biaxial plots and sequential analysis of pairs of markers, the t-SNE map captured and summarized the interrelationship between the 37 markers in an unbiased, data-driven manner. Consequently, t-SNE enabled the unbiased identification of new immune subsets by clustering the cells with previously unknown patterns of marker expression. By this method, we observed a clear difference in CD4^+^ and CD8^+^ T cell subsets between the 2 disease groups (RE and AIE) and healthy controls (HC) (Figure 1A, map regions 1–3). While the distribution of CD4^+^ and CD8^+^ T cells was similar between the AIE and the RE patients, the distribution of NK cells differed, with a lower frequency of NK cells in AIE compared with RE or HC (Figure 1A, map region 4). To identify the cellular phenotypes, we overlaid cell marker expression in color on the t-SNE map (Supplemental Figure 2). This expression overlay on the 2-D t-SNE map showed groupings of the cells by lineage, trafficking, and functional markers (Supplemental Figure 2). These groupings were further validated by manual gating and overlay on t-SNE coordinates in FlowJo software (Supplemental Figure 3). Concordance between manual FlowJo gating and clustering of major lineage cells by t-SNE validated the capability of the t-SNE algorithm to capture and summarize the CyTOF data on a 2-D t-SNE map.

### AIE and RE immune subsets share a dysregulated NI profile.

To identify potentially novel immune cell subsets in an unbiased manner, we clustered the cells using a k-means clustering algorithm. The number of clusters were determined using the Gaussian-means method (15). We analyzed the staining data obtained from the 2 antibody panels separately. K-means clustering grouped the cells stained with antibody panel 1 into 101 nodes and grouped the cells stained with antibody panel 2 into 135 nodes. We then identified the phenotypes of the nodes using the median expression value of surface and intracellular markers.

We hypothesized that the distribution of immune subsets might be unique to a pathophysiological condition and, thus, could differentiate HC from RE and AIE. To evaluate our hypothesis, we performed a correspondence analysis (CA) to summarize the data into lower dimensions and group the subjects with a similar immune subset distribution near to each other. The T cell and NK cell nodes from panel 1 and the B cell nodes from the panel 2 staining data were merged into a combined set of 106 nodes. The fraction of each node was calculated, and CA summarized the proportions of the 106 nodes in 2-D graphical form. CA clearly grouped HC into a very tight cluster that was clearly distinct from the RE and AIE groups. Conversely, we observed no clear distinction between the AIE and RE groups (Figure 1B). Clear separation of HC from the 2 disease groups suggests an altered balance of immune subsets in AIE and RE; the similarities in the AIE and RE immune profiles suggest a shared dysregulation and underlying NI mechanism.

To identify the dysregulated nodes, we used the nonparametric Wilcoxon rank-sum test to compare the mean frequency of nodes from the AIE and RE patients with the mean frequency of the nodes from HC. As the CA could not separate AIE from RE, we combined both disorders into 1 NI epileptic disease group and compared this group with HC. The Benjamini-Hochberg multiple test correction procedure was run to control for FDR. With a 10% FDR, we detected 49 nodes (Supplemental Table 5) that were significantly altered between the NI and HC groups, with 19 nodes expanded and 30 nodes contracted (Figure 1C).

### Proinflammatory IL-17^+^CD4^+^ T cell subsets expand in RE and AIE.

**Figure 2:**
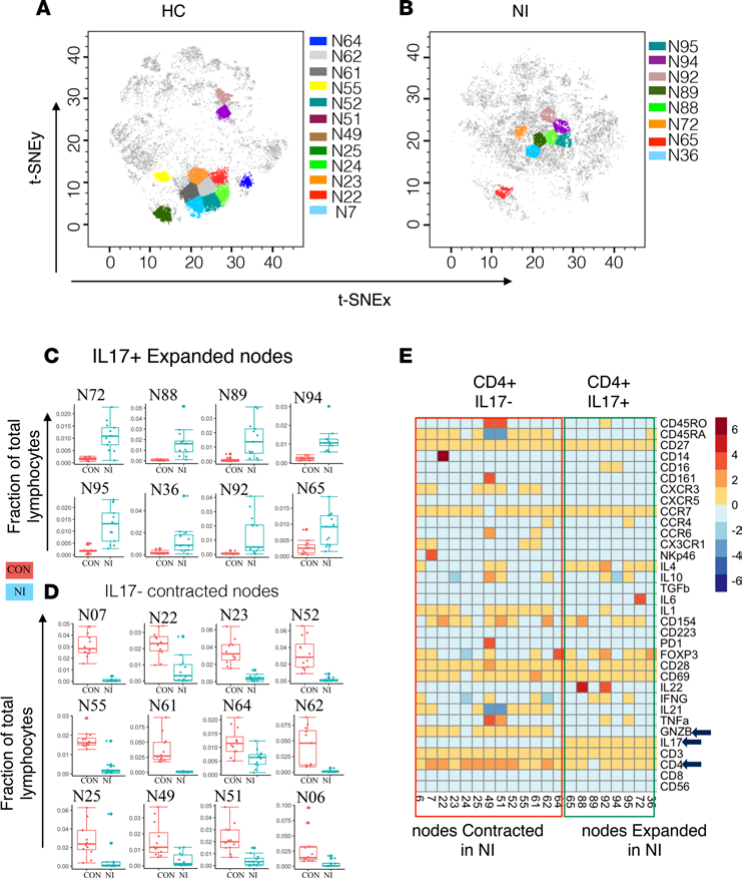
Proinflammatory IL-17–producing CD4^+^ T cell subsets expand in neuroinflammatory epileptic disease. (**A**) The position of CD4^+^IL-17^–^ nodes on the t-SNE map representing the healthy control (HC) group. CD4^+^IL-17^–^ nodes were contracted in the neuroinflammatory (NI) epileptic disease group (refractory epilepsy and autoimmune encephalitis) compared with the HC group. (**B**) The position of proinflammatory CD4^+^IL-17^+^ nodes on the t-SNE map representing the NI group. CD4^+^IL-17^+^ nodes were expanded in the NI group compared with the HC group. (**C**) The distribution of each expanded CD4^+^ node. (**D**) The distribution of each contracted CD4+ node. (**E**) The median expression value of surface or intracellular marker for each of the differentially modulated CD4^+^ nodes.

t-SNE enables dimensionality reduction, and visualization showed clear differences in the distribution of CD4^+^ T cell subsets between NI and HC (Figure 2, A–D). Clustering and differential node analysis confirmed a significant difference in CD4^+^ T cell nodes. By analyzing the surface and intracellular marker expression on the nodes (Figure 2E), we found that CD4^+^IL-17^+^ T cell subsets are expanded (Figure 2, B and C) and CD4^+^IL-17^–^ T cell are contracted in (Figure 2, A and D) NI compared with HC. This distribution clearly shows prominent proinflammatory IL-17 mechanisms. Although, these differentially modulated CD4^+^ T cell nodes were clearly IL-17^+^ or IL-17^–^, these nodes were heterogeneous in their functional marker expression. The expanded IL-17^+^ population was negative and the contracted IL-17^–^ population was positive for granzyme B (GZMB; Figure 2E). Among the expanded nodes, 2 were IL-22^+^ and 2 were both IFN-γ^+^ and TNF-α (Figure 2E). Interestingly, the expanded IL-17^+^CD4^+^ T cells were not enriched for CCR6 and CD161, contrary to what is often reported. We have evaluated CCR6 and CD161 expression by FlowJo and ruled out the experimental artifact. Indeed, we found that IL-17^+^CD4^+^ T cells from healthy individual were CCR6^+^ and CD161^+^; however, cells from NI patients were largely CCR6^–^ and CD161^–^ (Supplemental Figure 4). Hence, the CCR^–^ and CD161^–^ phenotype of the IL-17^+^CD4^+^ T population might be specific to disease condition.

### Inhibitory Lag3^+^CD8^+^ T cell subsets contract in RE and AIE.

**Figure 3:**
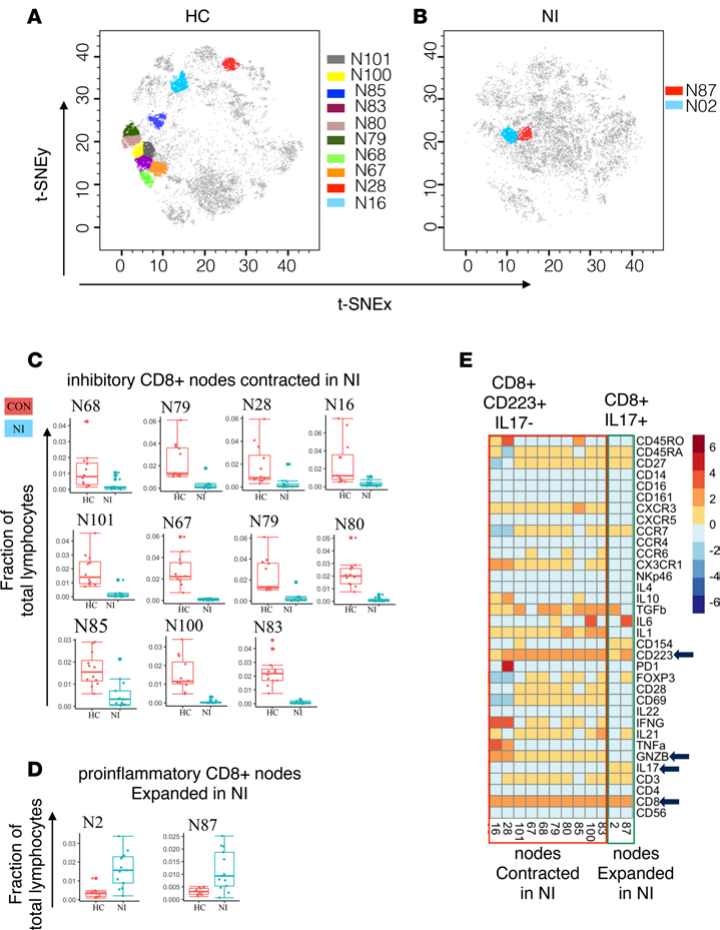
Inhibitory CD8^+^Lag3^+^ T cell subsets contract in patients with neuroinflammatory epileptic disease. (**A**) The position of CD8^+^Lag3^+^ nodes on the healthy control (HC) t-SNE map. (**B**) The position of proinflammatory CD8^+^IL-17^+^ nodes on the neuroinflammatory (NI) epileptic disease t-SNE map. (**C**) The distribution of markedly contracted CD8^+^ nodes. (**D**) The distribution of markedly expanded inhibitory CD8^+^ nodes. (**E**) The median expression value of surface or intracellular marker for each of the differentially modulated CD8^+^ nodes.

We next performed analysis of the CD8^+^ T cell nodes. We found that the majority of the differentially modulated CD8^+^ T cell nodes were contracted in the NI group compared with HC (Figure 3, A–C). The contracted CD8^+^ T cell nodes were all high in Lag3 (CD223) expression (Figure 3E), suggesting loss of inhibitory Lag3^+^CD8^+^ T cell subsets in RE and AIE. The 2 expanded CD8^+^ T cell nodes (nodes N02, N87) were IL-17^+^ (Figure 3, A–E) but CD28^–^ (Figure 3E). CD28 is a marker for antigen-induced stimulation; repeated TCR activation and presence of TNF-α and type-1 interferons increases the frequency of CD8^+^CD28^–^ T cells and indicates a proinflammatory status in autoimmune disease (16). Contraction of inhibitory Lag3^+^CD8^+^ T cells might help to amplify the inflammatory milieu created by these expanded proinflammatory CD4^+^ and CD8^+^ T cells by reducing the total number of cells capable of counterbalancing proinflammatory T cells.

### B cells with high Ig production potential expand in RE and AIE.

**Figure 4:**
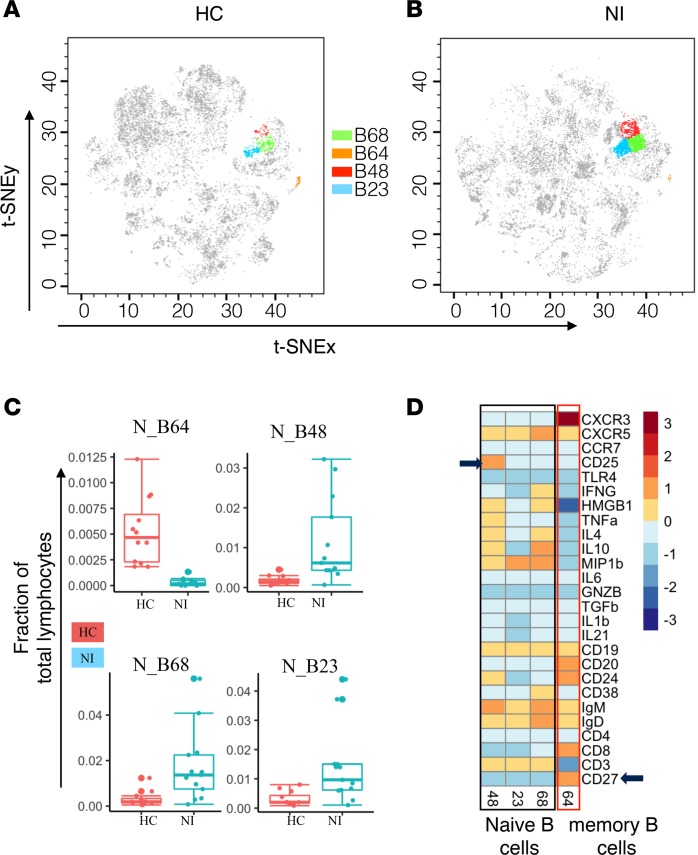
Imbalance in B cell subsets in patients with neuroinflammatory epileptic disease. (**A** and **B**) The position of CD19^+^ B cell nodes on the healthy control (HC) and neuroinflammatory (NI) epileptic disease t-SNE maps. (**C**) The distribution of markedly altered CD19^+^ B cell nodes. (**D**) Differentially modulated CD19^+^ B cell nodes phenotype represented as heatmap showing median expression markers.

For B cell analysis, we used a separate panel of antibodies (Supplemental Table 3), which included B cell markers. After clustering the cells, we identified 16 B cell nodes on the basis of CD19 expression. We then applied the nonparametric Wilcoxon rank sum test to evaluate the difference in mean frequency of the nodes between the HC and NI groups. Of the 16 nodes, 4 nodes were significantly different (10% FDR) in NI compared with HC (Figure 4, A–C). Specifically, the frequency of node 64 was reduced in NI, while the frequency of nodes 48, 68, and 23 was increased in NI (Figure 4, A–C). Node 64 had an IgM^+^ memory phenotype (CD27^+^IgD^+^IgM^+^), whereas nodes 48, 68, and 23 had a CD27^–^ phenotype (Figure 4D). These data suggest that the nodes expanded in AIE and RE groups have a naive phenotype (Figure 4D). In addition, node 48 was positive for CD25 surface expression (Figure 4D). CD25^+^ B cells have higher Ig production and enhanced antigen presenting ability (17). An increase in circulating CD25^+^ B cells in AIE and RE might contribute to increased autoantibody production, leading to inflammation in AIE and RE.

### NK cell subsets show cytokine heterogeneity and elevated Fractalkine receptor expression in RE.

**Figure 5:**
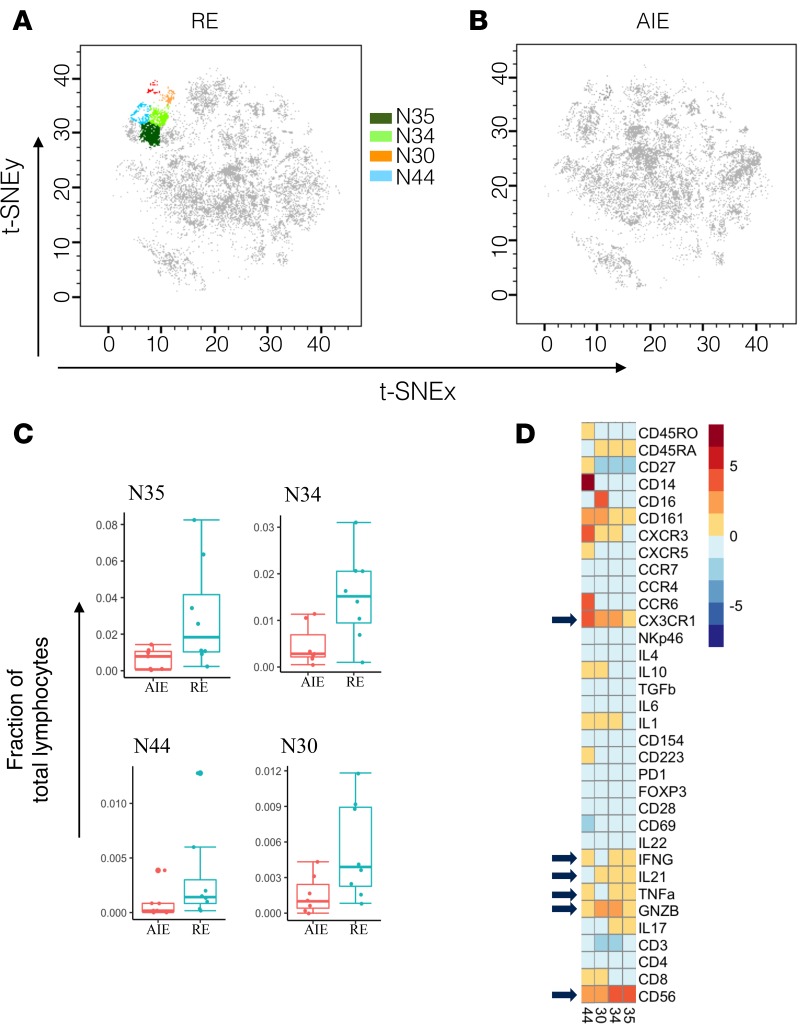
Fractalkine receptor–positive (CX3CR1^+^) NK cell subsets expand in refractory epilepsy (RE). (**A** and **B**) The position of CD56^+^ NK cell nodes on the RE and autoimmune encephalitis (AIE) t-SNE maps. (**C**) The distribution of markedly altered CD56^+^ NK cell nodes. (**D**) The median expression value of surface or intracellular marker for each of the differentially modulated CD56^+^ NK cell nodes.

Our initial dimensionality reduction and clustering using t-SNE showed differences in NK cell subset distribution between the AIE and RE groups (Figure 1A). We therefore tested whether NK cell subsets were significantly dysregulated in RE compared with AIE. Our unbiased, data-driven, CyTOF-assisted phenotyping grouped the NK cells into 10 subsets with heterogeneous phenotypes. Out of 10 nodes, 4 (nodes 35, 34, 44, 30) were significantly dysregulated (10% FDR) in RE compared with AIE (Figure 5, A and B), found at a markedly higher frequency as a proportion of total lymphocytes (Figure 5C). Node 44 showed high fractalkine receptor (CX3CR1) expression compared with the other 3 nodes (Figure 5D). This expansion in fractalkine receptor–positive, poly-functional NK cell subsets might contribute to persistent inflammation, leading to RE. All 4 dysregulated nodes were heterogeneous in their cytokine expression profile; nodes 34 and 35 were negative for IL-10 expression, whereas nodes 44 and 30 had low IL-10 expression (Figure 5D). Node 30 was negative for TNF-α and IFN-γ, whereas nodes 44, 34, and 35 were positive for TNF-α and IFN-γ (Figure 5D). Such heterogeneity in cytokine production suggests modulation of specialized NK cell functions.

### Architectural change and loss of negatively regulated intercellular connections characterize the RE and AIE immunome network.

**Figure 6:**
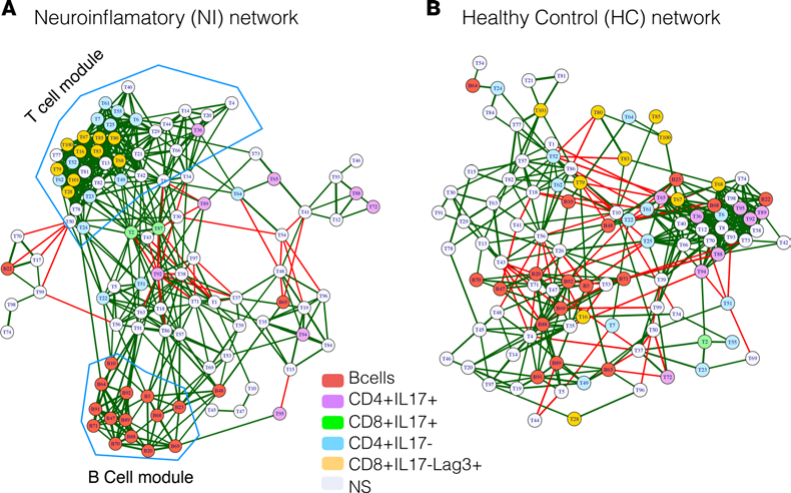
Coregulatory network of immune subsets in neuroinflammatory epileptic disease shows loss of negative regulatory edges and a highly modular structure. Networks of nodes (cellular subsets) were created and plotted as force-directed layout. The color of the nodes indicates the cellular phenotypes: B cells, red); markedly altered CD4^+^IL-17^+^, purple; CD4^+^IL-17^–^,cyan; CD8^+^IL-17^+^, green; CD8^+^Lag3^+^ T cell nodes, yellow; and nonsignificant nodes (NS), light gray. The edges (lines connecting 2 nodes) represent the connections between the nodes. The colors of the edges indicate the correlation direction: red, negative; green, positive. (**A**) Network of immune cell subsets (nodes) from patients with neuroinflammatory (NI) epileptic disease. (**B**) Network of immune cell subsets (nodes) from healthy controls (HC).

To analyze how immune cells interact and coordinate at the system level, we created a correlation network of cellular subsets (nodes). Here, we calculated the proportion of each node of total PBMCs, and the correlations among the cellular nodes were used to define the edges between the nodes in the cellular network. To create the network, 2 nodes were connected if the absolute correlation among them was >0.6. The correlation network was plotted using a force-directed Fruchterman-Reingold graph layout. The color of the edge reflects the direction of the correlation (red, negative; green, positive), while the edge thickness indicates the magnitude of the absolute correlation. Properties of the network were calculated and analyzed to investigate the immune cell communication at system level (Supplemental Table 4). The correlation network showed that the number of negatively correlated edges was reduced in the NI network (6.27%; Figure 6A) compared with the HC network (14.45%; Figure 6B). Furthermore, the NI network showed higher modularity (0.146; Figure 6A) compared with the HC network (0.087; Figure 6B). This higher modularity in the NI cellular network was evidenced by highly correlated, tightly clustered B cell and T cell subsets and modules (Figure 2A). Higher modularity among the cells of similar lineage suggests emergence of functions specific to disease state. Network analysis also revealed a higher centralization score for the NI cellular network (centralization score = 0.194) compared with the HC control network (centralization score = 0.139), indicating that the disease network is dominated by a few nodes and geared for more specific functions centralized around dominating nodes.

When we identified and color-coded the phenotypes of the differentially regulated nodes in the NI network, we found that the proinflammatory CD4^+^IL-17^+^ nodes were spread, whereas the inhibitory CD8^+^Lag3^+^ nodes clustered together. The opposite finding was observed in the HC cellular network (Figure 6). This spread of proinflammatory nodes in the NI network suggests an increase in intercellular communication between the IL-17^+^CD4^+^ nodes and nodes of other cellular origins in the NI network. At the same time, the clustering of inhibitory nodes in NI network suggests a break in intercellular communication and loss of regulatory mechanisms. Taken together, the expansion in proportion and spread of proinflammatory nodes, and the concomitant reduction in proportion and grouping of inhibitory nodes within the NI cellular network, suggest a loss of inhibitory mechanisms and emergence of proinflammatory functions in RE and AIE. Thus, our network analysis indicates that higher modularity, loss of negatively correlated interconnection, and higher centralization of the network leads to a loss of regulatory mechanisms and emergence of tightly regulated pathogenic functions in epileptic disorders.

## Discussion

In the present study, we investigated the immunome of pediatric patients with RE and AIE at the single-cell level using CyTOF. Our unbiased, data-driven analysis identified many unknown dysregulated immune cell subsets in RE and AIE compared with HC. t-SNE dimensionality reduction and clustering detected a cellular distribution pattern that was specific to HC, RE, and AIE patients (Figure 1A), with modulation in the CD4^+^ T cell, CD8^+^ T cell, and NK cell compartments.

Our CA clearly indicated a homogeneous immune response in HC that is distinct from the immune response in RE and AIE patients (Figure 1B). The inability of CA to segregate those with AIE from those with RE suggests that largely similar immune mechanisms underlie both disease groups and that persistence of these mechanisms may contribute to the development of RE.

We used automated clustering to group the cells with similar phenotypes and detect potentially novel immune subsets. This approach allowed us to uncover immune subsets with heterogeneous surface and functional marker expression within the previously known immune cell subsets. Within the T cell compartment, we identified several nodes with heterogeneous functions that were dysregulated to tip the overall balance toward proinflammatory IL-17 production by both CD4^+^ and CD8^+^ T cell subsets. This proinflammatory condition is aggravated by the expansion of IL-17–producing CD4^+^ and CD8^+^ T cells (Figure 2C and Figure 3D) and simultaneous reduction in inhibitory CD4^+^ and CD8^+^ subsets in RE (Figure 2D and Figure 3C).

A role for IL-17 in the pathogenesis of various NI diseases has been described (18–22). Patients with epilepsy exhibit higher IL-17 serum and CSF levels compared with HC, and this phenotype is associated with seizure severity (23). IL-17 helps permeabilize the human blood-brain barrier to soluble molecules and circulating CD4^+^ lymphocytes, and GZMB^+^ Th17 cells exert cytolytic activity on human fetal neurons (18). A recent study using the kainic acid mouse model of status epilepticus found a direct effect of IL-17 on neuronal excitability and cell viability, as well as delayed seizure onset in IL-17RA–deficient mice (8). In our study, we found that the expanded IL-17 T cell subsets in patients with RE did not express GZMB (Figure 2E). This lack of GZMB suggests that seizures may be precipitated by the proinflammatory function of IL-17 cytokines rather than as a direct result of IL-17^+^GZMB^–^ T cell cytolytic activity on neuronal cells. Conventionally, IL-17–producing T cells have been reported to express CCR6 and CD161; however, expanded IL-17^+^CD4^+^ T cells from NI were not enriched for these surface markers. In autoimmune disease, expansion of the CD146-enriched IL-17^+^ T cell population has been reported, while no or very low correlation between IL-17 and CCR6, CD161, or CCR2 was found (24). These pattern suggests that this heterogenous Th-17 T cell population is specific to disease condition. Together, permeabilization of the blood-brain barrier and increased neuronal excitability by IL-17 may explain the expansion of IL-17–producing T cells in seizure and epilepsy patients.

Proinflammatory mechanisms are counterbalanced by inhibitory mechanisms, and the shift in inhibitory/proinflammatory mediators leads to a pathological state. Inhibitory receptors prevent inflammation, autoimmunity, and tissue damage by dampening costimulatory receptor activity and limiting T cell activation. Lag3 (CD223) and PD1 are 2 well-known inhibitory receptors (25–27). In our RE cohort, we observed contraction of the inhibitory Lag3^+^CD8^+^ T cell subsets compared with HC. Among these contracted T cell subsets, node 28 (CD8^+^PD1^+^) and node 49 (CD4^+^PD1^+^) were also positive for the PD1 inhibitory receptor. Together, these results suggest that the balance between the proinflammatory and inhibitory mechanisms is highly skewed toward a proinflammatory state in RE.

B cells have been implicated in epilepsy since the detection of neuronal autoantibodies in the serum and CSF of patients with AIE and epilepsy (28–30). However, the presence of neuronal autoantibodies in just 4%–10% of epilepsy patients (29) and its poor association with course of epilepsy undermines the role of B cells in the pathogenesis of seizures and epilepsy. Apart from neuronal autoantibody production, B cells might influence neuroinflammation by regulating proinflammatory T cells. Indeed, we discovered an altered balance of B cell subsets in patients with RE and AIE compared with HC. Specifically, we observed a reduction in the IgM^+^CD27^+^ memory B cell subset (node 64) in patients with AIE and RE compared with HC. Circulating IgM memory B cells are generated by T cell–independent mechanisms, and they are the first line of defense against infections (31, 32). Contraction in the IgM memory B cell population in AIE may prolong the infection period and, hence, the inflammatory response to infection. Contraction of the IgM^+^ memory B cell population also in RE reinforces the similarity in the underlying pathogenic mechanisms of RE and AIE. Among the expanded B cell subsets, CD25^+^ subsets (node 48) may further aggravate inflammation due to enhanced antigen-presenting ability and higher levels of Ig production (17, 33).

Previous efforts to identify individual contributors of epilepsy pathogenesis and persistence have used conventional analytical frameworks in immunology. However, newer technologies such as CyTOF make it possible to perform deep analysis of the immune system, vastly improving our understanding of the intricacies of the immune response at a systems level. Such systems-level analyses have the capacity to identify the most relevant components of the immune system that can lead to pathogenesis and can provide insights into the optimal targets to alleviate the pathogenic response. Here, we built a correlation network of immune cell subsets to understand the intercellular dynamics within the immune system in NI patients compared with HC. We utilized approaches from social network analysis to better understand the overall immune cell network. Our network analysis results demonstrated modulation of the cellular networks in the NI group (RE plus AIE) (Figure 6A). Specifically, we found a higher modularity, loss of negatively correlated interconnection, and higher centralization score in the NI immune network compared with HC. These data imply loss of regulatory mechanisms and emergence of tightly regulated pathogenic functions in the NI group. We propose that a network analysis approach might help prognosticate epilepsy early in the course of the disease and better predict the individual treatment response. Instead of investigating a specific cellular subtype in isolation, a systems-level analysis of the immunome can provide an accurate assessment of the overall immune system before and after therapeutic intervention.

Seizures in AIE generally improve after the initial acute phase; however, a proportion of patients subsequently develop RE (10%–15 %) (2). Biomarkers are urgently needed to predict disease severity and the risk of developing RE, which in turn could guide treatment intensity and improve prognosis in AIE. Our t-SNE map of the AIE and RE groups indicated a difference in NK cell distribution (Figure 1A), and subsequent statistical analyses of the NK cell subsets uncovered a significantly higher frequency of fractalkine receptor (CX3CR1) positive proinflammatory subsets in RE compared with AIE (Figure 5, C and D). These NK cell subsets are multifunctional and produce GNZB, TNF-α, IFN-γ, and IL-21 (Figure 5D); they might exert direct cytolytic activity by GNZB or affect neuron and astrocyte function via proinflammatory cytokine release. The NK subsets might traffic to the CNS via the fractalkine ligand **—** a chemoattractant produced by neurons and astrocytes (34, 35). Once in the CNS, they may exacerbate the proinflammatory condition in the CNS directly via fractalkine ligand–receptor interactions with neuronal cells or indirectly by modulating the cellular functions of other immune cells in the peripheral circulation and body. Precision targeting of these NK cells presents a potential preventative strategy for epileptogenesis and development of RE.

In an ideal situation, concurrent analysis of the CSF in affected patients would confirm the link between inflammation in the peripheral blood and within the CNS. However, clinical considerations in the pediatric group precluded CSF analysis and limited the extent to which the relationship between these anatomical spaces could be measured. However, the traditional concept of a CNS isolated from the peripheral nervous system by the blood-brain barrier is certainly no longer valid. Many studies have established a link between the peripheral immune system and the CNS (36–38). In addition, a recent study using a humanized mouse model of Rasmussen’s encephalitis demonstrated trafficking of cellular subsets from the periphery to the CNS (8, 39); the study also demonstrated the trafficking of proinflammatory IL-17 and IFN-γ producing CD4^+^ T cells in mouse brain from the periphery, further substantiating the relevance of investigating the peripheral immune cells. Longitudinal follow-up of AIE cases and their progress to RE is now required to further pinpoint the role of specific immune cells in epileptogenesis. Further limitations of the study are the small sample size and all the samples are of Asian ethnicity. However, wholistic analytical approach all indicating emergence of proinflammatory mediators in epilepsy and seizure suggest that our finding probably will hold true in large cohorts and across ethnicities.

In conclusion, our comprehensive immune function analysis of leukocytes from pediatric patients with RE and AIE showed a similar immune profile that markedly differs from HC. We discovered that certain NK cell subsets are specifically overrepresented in RE compared with AIE. Our systems-level analysis showed modulation of the immune cell network and loss of regulatory mechanisms in the epileptic immunome. We observed systemic reduction in immune regulatory mechanisms, with contraction of T cells with inhibitory receptors and simultaneous expansion of proinflammatory cells, particularly IL-17–producing T cells. Together, our network and statistical analysis of immune cell subsets clearly demonstrate systemic dysregulation of the immunome that leads to a proinflammatory state in AIE and RE. We believe that our results and analytical framework have the potential to serve as a resource for the development of better diagnostic, prognostic, and therapeutic tools. A targeted immunotherapeutic approach holds the potential to improve treatment responses compared with untargeted immunotherapy. Biologics that target IL-17 are already undergoing clinical trials (40–42) for various autoimmune diseases. Repurposing these IL-17 blockers could facilitate early clinical trials for the prevention and treatment of RE.

## Methods

### Blood samples.

Peripheral blood samples were collected in EDTA acid tubes. Peripheral blood mononuclear cells (PBMCs) were isolated from blood samples layering over Ficoll-Paque Plus (GE Healthcare) and were stored in liquid nitrogen until further use. Full clinical and demographic information of the patients and samples used for various experiments is summarized in Supplemental Table 1.

### CyTOF.

Two panels of antibodies were designed and used for comprehensive immunome analysis: panel 1 antibodies focused on T cells and NK cells, while panel 2 focused on B cells (Supplemental Tables 2 and 3). To reduce the technical variability and simultaneous sample processing, a combination of 3 anti-CD45 antibody barcodes were used, as previously described (18). See Supplemental Tables 2 and 3 for complete list of antibodies and the vendors. The antibodies were either conjugated in-house according to the manufacturer’s instructions (Fluidigm) or purchased preconjugated directly from the supplier (Fluidigm). PBMCs were thawed and rested overnight in complete RPMI medium supplemented with 10% FBS, 1% penicillin/streptomycin/glutamine, and 10 mM HEPES at 37°C. RPMI, FBS, penicillin/streptomycin/glutamine, and HEPES were purchased from Gibco. The cells were then stimulated for 6 hours with 150 ng/ml phorbol myristate acetate (PMA) and 100 ng/ml ionomycin (MilliporeSigma), and they were exposed to 3 μg/ml Brefeldin A (eBiosience) and 2 μM monesin (BioLegend) during the final 4 hours of the incubation. Next, the cells were stained with cisplatin (Fludigm) to identify live/dead cells and incubated with metal-conjugated surface-membrane antibodies. The cells were then fixed in 1.6% paraformaldehyde and permeablized in 100% methanol to permit staining with intracellular metal-conjugated antibodies. Finally, the cells were labeled with an iridium-containing DNA intercalator before analysis on a CyTOF-II mass cytometer (Fluidigm).

The signal was bead normalized using EQ Four Element Calibration Beads (EQ Beads, 201078, Fluidigm), according to manufacturer’s instructions. The generated .fcs files were filtered for live/dead cells and DNA and then manually debarcoded using FlowJo software. The debarcoded files were then analyzed in the R programming environment (Version R-3.4). For t-SNE dimensionality reduction, each sample was down-sampled to 5,000 cells. The resulting cells were clustered into nodes using k-means clustering. Samples with total cell counts <5,000 were excluded from the analysis.

### Network analysis.

The proportion of each node was calculated for every patient, and the correlation between nodes was calculated for HC, RE, and AIE. To construct the network, nodes were connected if they had an absolute correlation coefficient >0.6. The network was visualized and analyzed using the igraph R package.

### Statistics.

The Wilcoxon test (coin R package) was used for differential node analysis. To control for FDR, *P* values were adjusted using the Benjamini-Hochberg multiple test correction. CA was performed using the CA function in the FactoMiner R package. *P* < 0.05 was considered statistically significant.

### Study approval.

Patients were recruited from the KK Women’s and Children’s Hospital. The cohort consisted of 10 pediatric patients with RE undergoing follow-up at the Epilepsy Clinic, 10 pediatric patients presenting with acute AIE, and 12 HC undergoing routine presurgical blood tests. The study was reviewed and approved SingHealth Central IRB. The Informed consent was obtained according to the SingHealth Central IRB requirements.

## Author contributions

PK performed the experiments and bioinformatics, analyzed the data, and wrote the manuscript. BP, AL, LLY, and SLP performed the experiments. SL and AN recruited the patients and obtained the relevant blood samples. TA and JGY recruited the healthy subjects and obtained the relevant blood samples. TA participated in manuscript preparation. SA and DCWS conceived the study, analyzed the data, and wrote the manuscript.

## Supplementary Material

Supplemental data

## Version 1 03/26/2019

In-Press Preview

## Version 2 04/18/2019

Electronic publication
