# Supplementary material for: Proinflammatory IL-17 pathways dominate the architecture of the immunome in pediatric refractory epilepsy
Source: JCI Insight. 2019 Apr 18;4(8):e126337. doi: 10.1172/jci.insight.126337 (PMC6538358; doi:10.1172/jci.insight.126337)
Supplement: Supplemental data [file jciinsight-4-126337-s063.pdf]

Supplemental Information

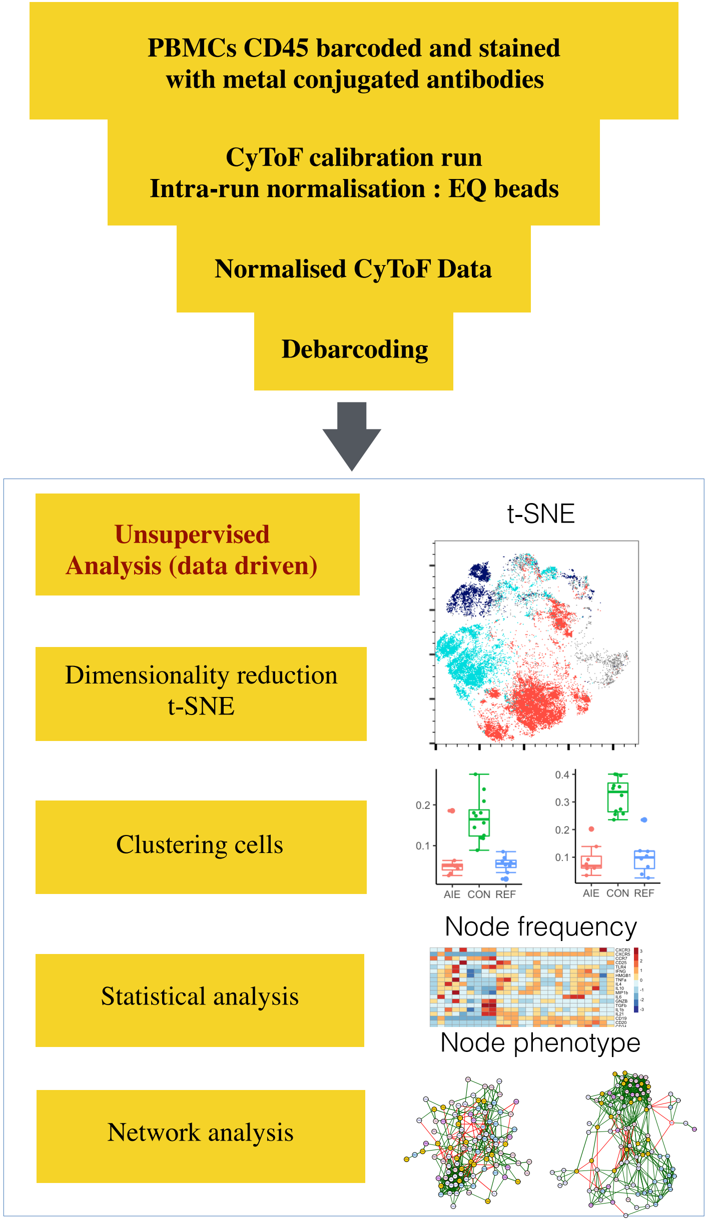

Supplementary figure1. Mass cytometry data acquisition and analysis workflow.

Experimental workflow to acquire and analyse the CyTOF data.

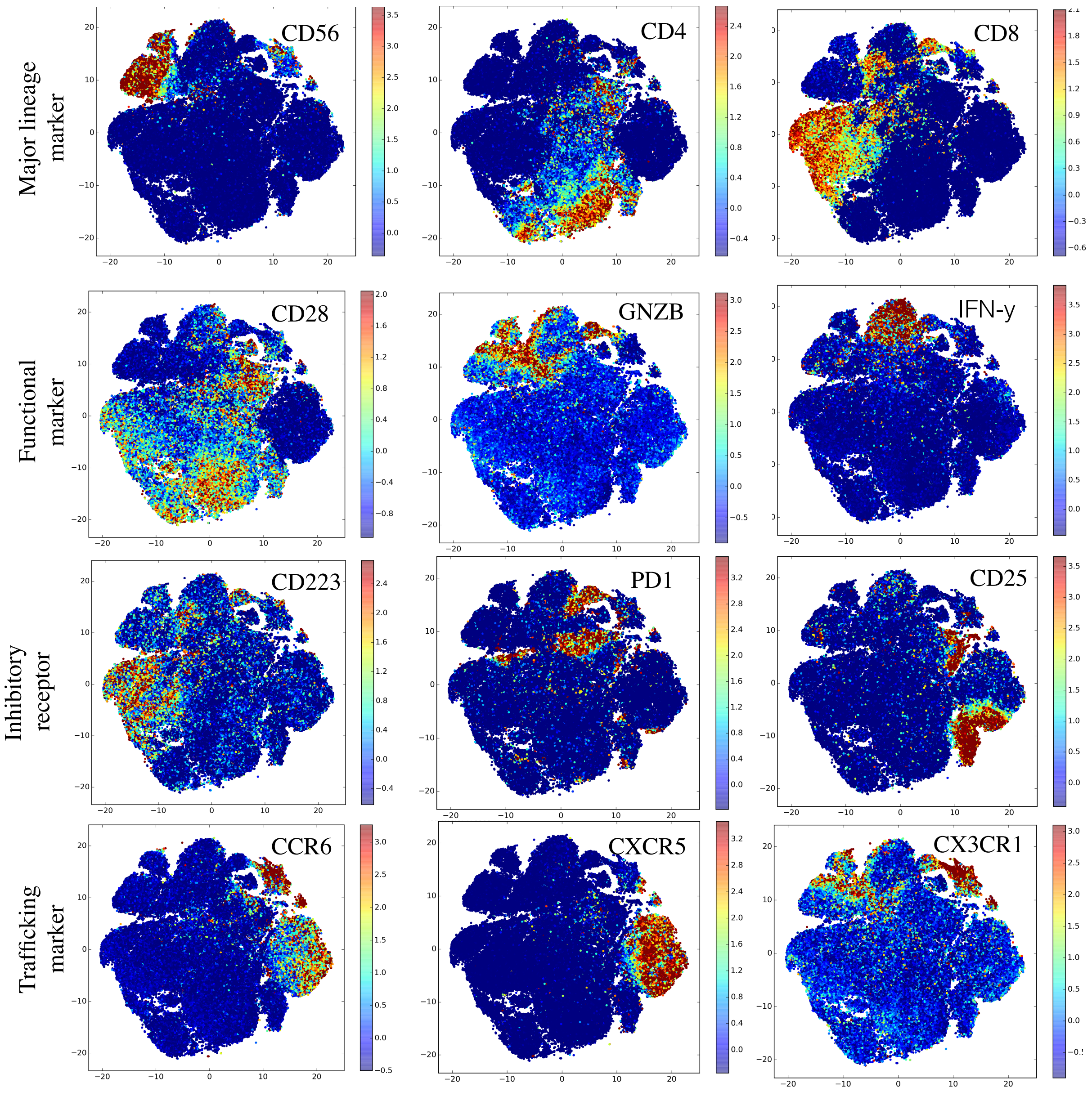

Supplementary figure 2. Global picture of immune cells visualised on a 2D t-SNE map.

The t-SNE summarizes the high dimensional CyTOF data on dimensional 2D t-SNE map. The t-SNE map captured and summarized the relationship between 37 markers in an unbiased, data-driven approach. The x and y axes indicate the t-SNE coordinates and the color indicates the expression of the overlaid markers. Plots were visualized in R programming using a custom script.

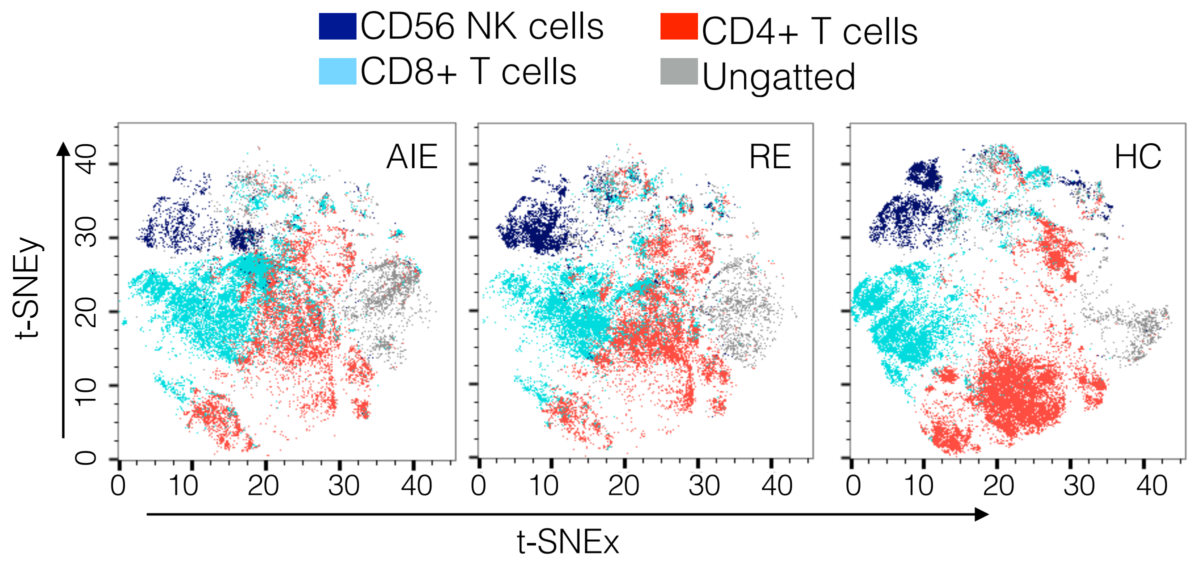

Supplementary figure 3. Distribution of major cell types visualized on a t-SNE map.
t-SNE maps with equal number for cells from each group were plotted for AIE, RE and HC. CD4+ T cells, CD8+ T cells and CD56 NK cells were gated by FlowJO software and the gated cells were overlaid on the t-SNE map. The color indicates the gated cell types. Plots were made using FlowJO software.

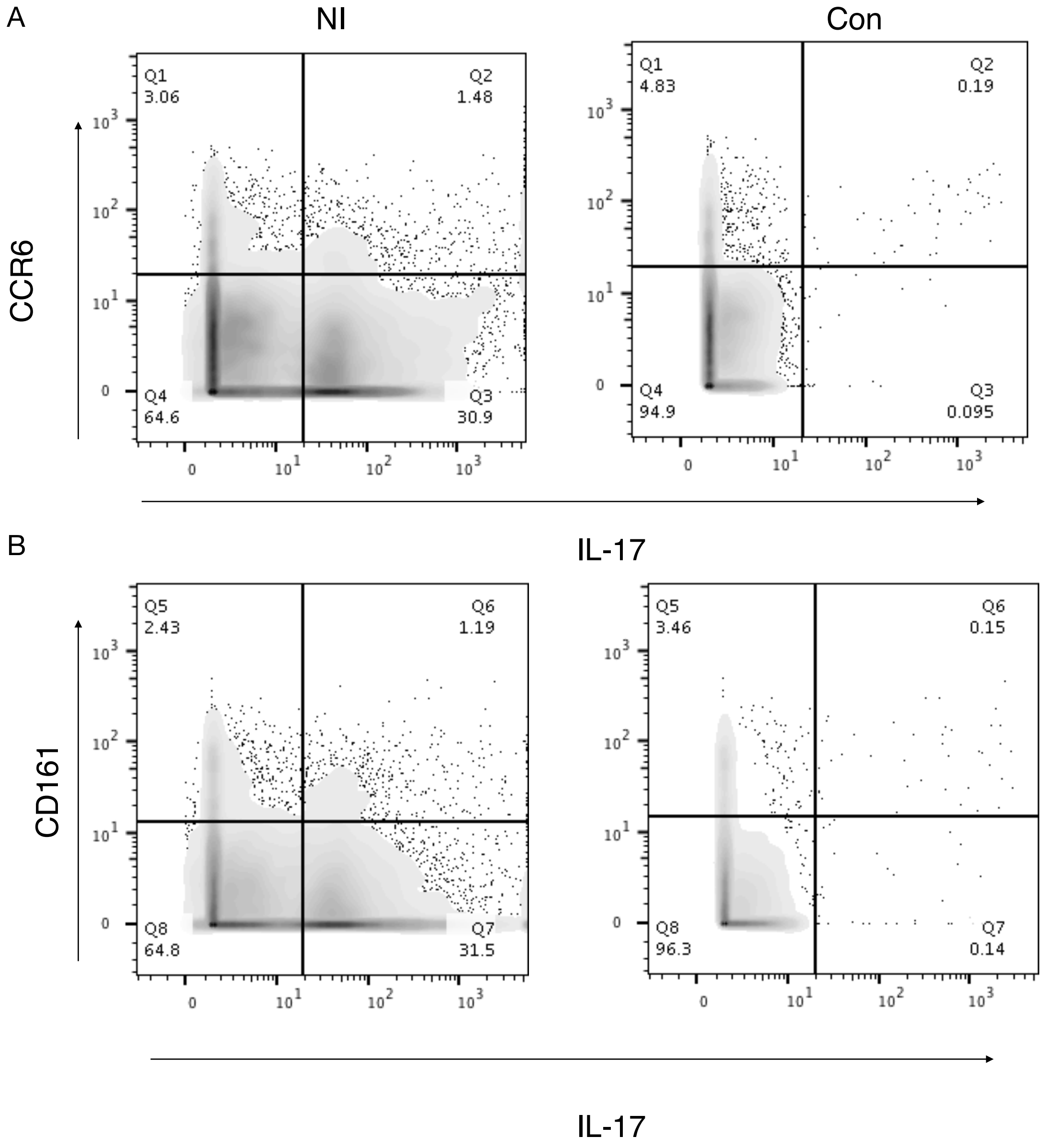

Supplementary figure 4. Expanded CD4+ IL-17+ T cell population from neuroinflammatory (NI) condition is largely CCR6 and CD161 negative.

Figure (A) shows CCR6 expression on y-axis while figure (B) shows CD161 expression on y-axis. X-axis shows IL-17 expression. To evaluate the percentage of IL-17+ cells quadrant gate was applied using flow software. Panel shows gated CD4+ T cells from NI (n=16) and Con (n=12)

Supplementary Table 1: Study subject and demographics and clinical characteristic of the patients.

SampleId	group	Gender	Age	Race	Diagnosis	Seizure onset	Seizure Semiology / Frequency	Freq per month Convulsive/ Tonic (MC/ absence/ spasms)	EEG	MRI	Medication	Aetiology, Comments
AIE_001	AIE	M	1.17	Chinese	AE	No seizures	NA	NIL	Not done	Bilateral deep grey, cerebral peduncle, cerebellar oedema	IMT	autoantibody screen negative
AIE_002	AIE	M	4.5	Indonesian	AE	4y	BC, Focal motor 3-4x/week	10-30	Generalized slowing	Normal	LVT, IMT	NMDAR antibody positive
AIE_003	AIE	F	8	Indonesian	AE	8y	Focal motor, BC 1x/month	< 10	Bi-temporal slowing	Arachnoid cyst, ventriculomegaly	VP	Negative serology
AIE_004	AIE	F	10.67	Malay	AE	No seizures	NA	NIL	Not done	T2 hyperintensity with restricted diffusion in left frontoparietal lobes	IMT	VGKC antibody positive
AIE_006	AIE	M	11.17	Chinese	AE	11y	TS 3x/week	10-30	Generalized slow waves	Bilateral symmetrical enlargement and T2 hyperintensity of BG with areas of thickened cortex	LVT, CLZ, IMT	autoantibody screen negative
AIE_008	AIE	M	12.75	Indian	AE	13y	BC, HC 12x/month	10-30	Slowing over temporal regions, then R frontal/ posterior discharges	Normal	PHB, LVT, TPM	autoantibody screen negative
AIE_009	AIE	M	1.17	Malay	AE	16m	SE, TS (6x/ day)	90-180	Generalized spike- wave discharges	Cerebral atrophy	LVT, PMP VP, TPM, PHB, CLZ, IMT	FIRES

AIE_010	AIE	F	15.75	Burmese	AE	No seiz ures	NA	NIL	Not done	Normal	IMT	autoantibody screen negative
REF_001	REF	M	1.33	Burmese	Focal epilepsy	3m	Tonic, Bilat. convulsive 20x/day	>180	R parieto-occipital epileptic discharges	Heterotopia adjacent to right occipital horn	LVT, CBZ, CLZ, ZNS, VGB, PHB	Focal cortical dysplasia Epilepsy surgery right parieto-occipital region at 16m
REF_002	REF	F	8.5	Chinese	Focal epilepsy	6y	Focal motor, Epileptic spasms 10-15x/m	10-30 (several daily)	Normal interictal	Encephalomalacia in the BG, cerebral and cerebellar atrophy	LVT	Leigh's syndrome Progressive neurodegeneration with dystonia, polymyoclonus and seizures
REF_003	REF	M	19.33	Chinese	Progress ive Myoclon ic Epilepsy	10y	Bilateral convulsive, Myoclonic, 1x/every 2 to 3m	<10 (several daily)	Polyspikes in central region	Normal	PCT, ZNS, LVT, VP	*KCNC1* mutation identified
REF_004	REF	F	20.83	Malay	Epilepsy with generali zed seizures	11y	Bilateral convulsive, tonic, absence Up to 3 x/ week	10-30 (several daily)	Anterior dominant generalized spike waves	Normal	LMT, LVT, ACZ, PHT, VP, ETX, CBZ	
REF_005	REF	F	8.5	Indian	Focal epilepsy	7m	Tonic, drop attacks	60-90	Multifocal discharges	Multiple subcortical tubers and subependymal nodules	CBZ, LTG, LVT, VP, CBZ, TPM, VGB, PMP	Tuberous sclerosis FDG PET scan and EEG non-localising

REF_006	REF	F	20.17	Indian	Progressive Myoclonic Epilepsy	3y	Bilateral convulsive Myoclonic 2-3 x per day	60-90 (several daily)	Generalized spike-wave discharges	Normal	PCT, VP, ZNS, NTZ, LMT, CLZ, LVT, ACZ, CLNZ, pyridoxine, IMT	
REF_007	REF	M	2	Chinese	Focal epilepsy	5m	Tonic, Bilat. convulsive, Myoclonic 3x/day	60-90	Normal interictal EEG	Normal	CBZ, LVT, PMP	
REF_010	REF	F	12.67	Chinese	Progressive Myoclonic Epilepsy	10y	Bilateral convulsive, Myoclonic, Daily	30-60 (several daily)	Central ED	Normal	PCT, VP, PMP, LVT, ZNS	*KCNC1* mutation identified
CON_001	con	M	11	Malay	Healthy controls							
CON_002	con	M	9	Malay	Healthy controls							
CON_003	con	M	6	Indian	Healthy controls							
CON_004	con	M	6	Malay	Healthy controls							
CON_005	con	M	6	Chinese	Healthy controls							
CON_006	con	M	8	Chinese	Healthy controls							
CON_007	con	M	11	Indian	Healthy controls							

CON_008	con	F	13	Chinese	Healthy controls							
CON_009	con	F	6	Chinese	Healthy controls							
CON_010	con	F	12	Malay	Healthy controls							
CON_011	con	M	9	Malay	Healthy controls							
CON_012	con	M	13	Chinese	Healthy controls							

Medications : ACZ: Acetazolamide; CBZ: Carbamazepine, CLNZ: Clonazepam; CLZ: Clobazam, ETX: Ethosuximide; LVT: Levetiracetam; LMT: Lamotrigine; NTZ: Nitrazepam; OCBZ: Oxcarbazepine; PCT: Piracetam; PHB: Phenobarbitone; PMP: Perampanel; TPM: Topiramate; VGB: Vigabatrin, VP: Valproate; ZNS: Zonisamide

Supplementary Table 2. Panel 1 Antibodies conjugated metal, clone and vendor information

Metal	Antibody	Clone	Vendor
89	CD45A	HI30	Fluidigm
112/114	CD14	TüK4	Lifetechnologies
115	CD45B	HI30	Biolegend
139	CD3	145-2C11	Biolegend
141	CD28	37.51	Biolegend
142	CD45RO	UCHL1	Biolegend
143	IL-6	MQ2-13A5	Biolegend
144	CD8	SK1	Biolegend
145	TGF-b	TW4-6H10	Biolegend
146	TCR-gd	B1	Biolegend
147	PD-1	EHI12.2H7	Biolegend
148	CD4	SK3	Biolegend
149	biotin (IL-1beta)	HIb-98	Biolegend
150	PE-(IL-22)	2G12A41	Biolegend
151	TNF-a	MAb11	Biolegend
152	NKp46	9E+02	Biolegend
153	CD25	3C7	Biolegend
154	CD27	O323	Biolegend
155	CCR7	G043H7	Biolegend
156	IL-4	11B11	Biolegend
157	CCR4	L291H4	Biolegend
158	IL-10	JES3-9D7	Biolegend
159	CD45C	HI30	Biolegend

160	CD223	11C3C65	Biolegend
161	CD56	NCAM16.2	BD bioscience
162	CD62L	DREG56	Biolegend
163	CXCR3	G025H7	Biolegend
164	CD161	HP-3G10	Biolegend
165	FoxP3	PCH101	Ebioscience
166	CXCR5	J252D4	Biolegend
168	IFN-y	B27	Biolegend
169	IL-17A	BL168	Biolegend
170	CCR6	G034E3	Biolegend
171	CD45RA	JS-83	Ebioscience
172	IL-21	3A3-N2	Biolegend
173	GranzymeB	16G6	Biolegend
174	CX3CR1	K0124E1	Biolegend
175	CD154	24-31	Biolegend
176	CD69	FN50	Biolegend
209	CD16	3G8	Fluidigm
191/193	DNA Ir Intercalator		Fluidigm

Supplementary Table 3. Panel 2 Antibodies conjugated metal, clone and vendor information

Metal	Antibody	Clone	Vendor
89	CD45A	HI30	Fluidigm
112/114	CD14	TüK4	Lifetechnologies
115	CD45B	HI30	Biolegend
139	CD3	145-2C11	Biolegend
141	CD19	HIB19	Biolegend
142	CD45RO	UCHL1	Biolegend
143	IL-6	MQ2-13A5	Biolegend
144	CD8	SK1	Biolegend
145	TGF-b	TW4-6H10	Biolegend
146	IgD	11.26c.2a	Biolegend
147	CD20	2H7	Biolegend
148	CD4	SK3	Biolegend
149	biotin (IL-1beta)	HIb-98	Biolegend
150	HLADR	L-243	Biolegend
151	TNF-a	MAb11	Biolegend
152	CD24	MI/69	Biolegend
153	CD25	3C7	Biolegend
154	CD27	O323	Biolegend
155	CCR7	G043H7	Biolegend
156	IL-4	11B11	Biolegend
157	CD11c	3.9	Biolegend
158	IL-10	JES3-9D7	Biolegend
159	CD45C	HI30	Biolegend

160	IgM	MHM88	Biolegend
161	CD11b	ICRF44	BD bioscience
162	IL-8	BH0514	Biolegend
163	CXCR3	G025H7	Biolegend
164	CD161	HP-3G10	Biolegend
165	CD103	Ber-ACT8	Biolegend
166	CXCR5	J252D4	Biolegend
167	CD38	clone 90	Biolegend
168	IFN-y	B27	Biolegend
170	HMGB1	3E+08	Biolegend
171	CD45RA	JS-83	Ebioscience
172	IL-21	3A3-N2	Biolegend
173	GranzymeB	16G6	Biolegend
174	TLR4	HTA125	Biolegend
175	MIP-1beta	24006	R&D Systems
176	CD69	FN50	Biolegend
209	CD16	3G8	Fluidigm
191/193	DNA Ir Intercalator		Fluidigm

Supplementary Table 4: Properties of NI and HC network

Network property	NI Network	HC network
Modularity score	0.146	0.087
Network centralization	0.194	0.138
Network density	0.101	0.076
Network heterogeneity	0.584	0.690
Avg no of neighbours	10.323	7.924
Negatively correlated edges	34	60
Positively correlated edges	508	355

Supplementary Table 5. Node phenotype

Node	Marker Expression	Phenotype
6	CD4+CCR7+CD45RA+	Naive/effector CD4 T cells
7	CD4+CCR7+CD45RA+	Naive/effector CD4 T cells
2	CD4+CCR7+CD45RA+	Naive/effector CD4 T cells
23	CD4+CCR7+CD45RA+	Naive/effector CD4 T cells
24	CD4+CCR7+CD45RA-	Memory CD4 T cells
25	CD4+CCR7+CD45RA+	Naive/effector CD4 T cells
49	CD4+ PD1+	Inhibitory CD4 T cells
51	CD4+ CD45RA	Memory CD4 T cells
52	CD4+CCR7+CD45RA+	Naive/effector CD4 T cells
55	CD4+CCR7+CD45RA+	Naive/effector CD4 T cells
61	CD4+CCR7+CD45RA+	Naive/effector CD4 T cells
62	CD4+CCR7+CD45RA+	Naive/effector CD4 T cells

64	CD4+CCR7+CD45RA-	Memory CD4 T cells
65	CD4+ IL-17+	Th17
88	CD4+ IL-17+IL22+	Th17
89	CD4+ IL-17+IFNg+	Th17
92	CD4+ IL-17+IL22+IFNg+	Th17
94	CD4+ IL-17+	Th17
95	CD4+ IL-17+IFNg+	Th17
72	CD4+ IL-17+	Th17
36	CD4+ IL-17+	Th17
16	CD8+LAG3+CCR7-	
28	CD8+LAG3+PD1+	Memory CD8 T cells
101	CD8+LAG3+CCR7+CD45RA+	Naive/effector CD4 T cells
67	CD8+LAG3+CCR7+CD45RA+	Naive/effector CD4 T cells
68	CD8+LAG3+CCR7+CD45RA+	Naive/effector CD4 T cells
79	CD8+LAG3+CCR7+CD45RA+	Naive/effector CD4 T cells
80	CD8+LAG3+CCR7+CD45RA+	Naive/effector CD4 T cells
85	CD8+LAG3+CCR7-CD45RA-	Memory CD8 T cells
100	CD8+LAG3+CCR7+CD45RA+	Naive/effector CD4 T cells
83	CD8+LAG3+CCR7+CD45RA+	Naive/effector CD4 T cells
2	CD8+LAG3+CCR7+CD45RA+	Naive/effector CD4 T cells
87	CD8+LAG3+CCR7+CD45RA+	Naive/effector CD4 T cells
48	CD19+CD27-CD25+	Naive B cells
23	CD19+CD27-CD25-	Naive B cells
68	CD19+CD27-CD25-	Naive B cells
64	CD19+CD27+CD25-	Memory B cells

44	CD56+CX3CR1+GNZB+IFNg+	Pro-inflammatory NK cells
30	CD56+CX3CR1+GNZB+IL21+	Pro-inflammatory NK cells
34	CD56+CX3CR1+GNZB+TNFa+IFNg+	Pro-inflammatory NK cells
35	CD56+CX3CR1+GNZB+TNFa+IFNg+	Pro-inflammatory NK cells
